# Beyond Infection: The Role of Secreted Viral Proteins in Pathogenesis, Disease Severity and Diagnostic Applications

**DOI:** 10.3390/cells14090624

**Published:** 2025-04-22

**Authors:** Luis Herrera-Moro Huitron, Víctor Javier Cruz-Holguin, José Manuel Ulloa-Aguilar, Luis Adrián De Jesús-González, Juan Fidel Osuna-Ramos, Mario Guzmán-Huerta, Mercedes Piedad de León-Bautista, Guadalupe León-Reyes, Julio García-Cordero, Leticia Cedillo-Barrón, Jorge Francisco Cerna-Cortes, Moisés León-Juárez

**Affiliations:** 1 Laboratorio de Virología Perinatal y Diseño Molecular de Antígenos y Biomarcadores, Departamento de Inmunobioquímica, Instituto Nacional de Perinatología, Ciudad de México 11000, Mexico; luis_5m5@hotmail.com (L.H.-M.H.); vic_cruise@hotmail.com (V.J.C.-H.); josemanuel7111@gmail.com (J.M.U.-A.); 2 Laboratorio de Microbiología Molecular, Departamento de Microbiología, Escuela Nacional de Ciencias Biológicas, Instituto Politécnico Nacional, Ciudad de México 11340, Mexico; jorgecerna1008@gmail.com; 3 Unidad de Investigación Biomédica de Zacatecas, Instituto Mexicano del Seguro Social, Zacatecas 98000, Mexico; luis.dejesus@cinvestav.mx; 4 Facultad de Medicina, Universidad Autónoma de Sinaloa, Culiacán 80019, Mexico; osunajuanfidel.fm@uas.edu.mx; 5 Departamento de Medicina Traslacional, Instituto Nacional de Perinatología, Ciudad de México 11000, Mexico; mguzmanhuerta@yahoo.com.mx; 6 Escuela de Medicina, Universidad Vasco de Quiroga, Morelia 58090, Mexico; dramercedespiedad@gmail.com; 7 Laboratorio de Enfermedades Infecciosas y Genómica (INEX LAB), Morelia 58280, Mexico; 8 Laboratorio de Nutrigenética y Nutrigenómica, Instituto Nacional de Medicina Genómica (INMEGEN), Ciudad de México 14610, Mexico; greyes@inmegen.gob.mx; 9 Departamento de Biomedicina Molecular, Centro de Investigación y de Estudios Avanzados del Instituto Politécnico Nacional (CINVESTAV-IPN), Ciudad de México 07360, Mexico; jugarcia@cinvestav.mx (J.G.-C.); lcedillo@cinvestav.mx (L.C.-B.)

**Keywords:** secretion of viral proteins, pathogenesis, diagnostic, biomarkers of severity and immune system evasion

## Abstract

Secreted viral proteins are crucial in virus–host interactions, as they modify the host microenvironment to promote infection. These secreted proteins could alter immune and inflammatory responses, allowing viruses to evade defense mechanisms such as cytotoxic T cell activation and antibody neutralization. Some secreted proteins mimic host molecules to suppress antiviral responses, making them valuable targets for antivirals and diagnostics. Notable examples include BARF1 from Epstein–Barr virus, associated with gastric cancer; vIL-10 from Epstein–Barr virus, which regulates immune responses and contributes to autoimmune diseases; NS1 from dengue virus, associated with vascular permeability and early diagnosis; and NSP4 from rotavirus as an enterotoxin, among others. The study of these proteins improves our understanding of viral pathogenesis and helps to develop innovative treatments for infectious and non-infectious diseases, taking advantage of the evolutionary adaptations of viruses. This review explores their impact on the infection cycle, disease progression, and key processes, such as cell cycle regulation, apoptosis, and cell signaling. Research on these proteins deepens our basic knowledge of virology and generates alternative methods for detecting biomarkers and creating more effective therapies, as well as implementing some emerging technologies, such as biosensors and plasmon resonance, for the diagnosis of viral diseases.

## 1. Introduction

During the viral replicative cycle, infected cells release or secrete both host molecules and some viral proteins that play an essential role in the interaction with the host, compared to structural proteins such as the capsid or virion. Secreted proteins interact with the host microenvironment and facilitate the completion of the infection cycle. In other words, viral secreted proteins are critical multifunctional tools that have evolved to manipulate some key biological processes, such as the host inflammatory response, the immune response, signaling pathways, and cellular homeostasis [1]. The secretion of these viral proteins allows viruses to evade host defense processes in the first instance by evading NK cells, neutralizing antibodies, and activating cytotoxic T cells. There is another mechanism that involves the secretion of proteins that mimic some host cell molecules and thus disrupt or interfere with crucial immune pathways to deactivate the antiviral response exerted by infected cells [2].

The study of secreted viral proteins is key to understanding how viruses bypass immune defenses and exacerbate diseases [3]. These proteins are promising targets for antiviral therapies and vaccines, and can act as markers for early diagnosis, as they interact with host cells and are detectable in biological fluids. Notably, these molecules can also modulate the immune system, indicating their potential as tools to control inflammation or suppress autoimmunity. Beyond infections, these proteins inspire treatments for non-infectious diseases such as cancer and chronic inflammation by revealing evolutionary mechanisms perfected by viruses [3]. Applying this knowledge in clinical practice has enabled the development of precise, targeted therapies. This review describes some of the most studied secreted proteins, such as BARF1 and vIL-10 of Epstein–Barr virus, NS1 of dengue virus, NSP4 of rotavirus, Tat of HIV, the glycoproteins of RSV, and NSP2 and ORF8 of SARS-CoV-2, highlighting their involvement in immune response modulation, the alteration of cell signaling pathways, and pathogenesis, all of which are key processes that contribute to further pathogenesis during disease. Understanding these mechanisms is critical for the development of new therapeutic and antiviral strategies [4].

## 2. Exploiting the Cellular Machinery: Pathways Used by Secreted Viral Proteins

Secreted viral proteins are important factors in pathogenesis, which can help the virus evade the immune system, alter host response, and propagate. In some cases, these proteins act as decoys by binding to antibodies or cell receptors, reducing viral neutralization, inhibiting complement activation, and decreasing the host’s ability to control infection. For these proteins to be secreted, they usurp some molecules that participate in different cellular pathways. Extracellular vesicles or exosomes of endosomal origin play an important role, as they not only transport molecules, such as nucleic acids, metabolites, lipids, and proteins to target cells, but have also been shown to package and transport viral proteins, such as the NSP2 protein of SARS-CoV-2, the NS1 protein of DENV, and the BARF1 protein of Epstein–Barr virus, which hijack the exosomal pathway to be secreted [5,6,7,8]. It has been observed that certain viral infections can modulate exosome biogenesis, increasing the number of vesicles in infected cells. This phenomenon could be related to the internalization of viral proteins within the vesicles; for example, in the case of SARS-CoV-2, it has been shown that infected cells release exosomes containing viral components, suggesting that the virus can hijack the extracellular vesicle biogenesis machinery to facilitate its spread and evade immune detection [7,8,9]. In addition, during infection, dengue virus (DENV) secretes exosomes containing viral proteins and RNA, contributing to virus shedding and the modulation of the immune response [5,10,11,12,13].

**Figure 1:**
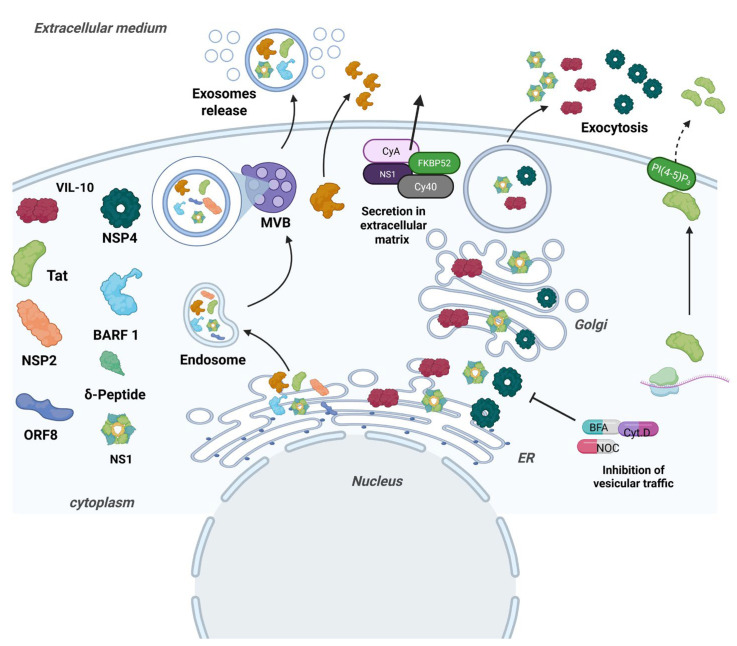
Pathways used by viral proteins for their release into the extracellular matrix. To be released into the extracellular medium, viral proteins occupy various pathways; however, it is known that the most commonly used are related to exocytosis, which basically means that vesicles containing proteins fuse with the cell membrane to release their contents. Additionally, they can also occupy the extracellular vesicle-dependent pathway, which includes the endoplasmic reticulum and the Golgi complex. Finally, another route that they can use is the interaction with elements of the caveolin complex and the interaction with phospholipids such as PI(4,5) P_2_. Once secreted, these proteins have some particular functions: for example, vIL-10 acts on neighboring immune cells, and its main function is to inhibit the production of proinflammatory cytokines, which reduces inflammation; Tat protein, when released into the extracellular environment, acts on neighboring cells, and it specifically affects the production of cytokines and immune mediators; 0RF8 in the extracellular medium can decrease the expression of MHC-I to interfere with the antiviral immune response; and by being secreted, NS1 fulfills the function of modulating the immune response by affecting vascular permeability. The NS1 protein is secreted in a classic way, and when in the extracellular medium, it can alter the vascular permeability; BARF1, once secreted, can stimulate cell proliferation and modulate the immune response; NSP4, once secreted, performs an enterotoxin function, by altering the calcium homeostasis of intestinal cells.

Rab proteins, which are a subfamily of GTPases, oversee vesicular trafficking and control the formation, transport, anchoring, and fusion of vesicles in different cellular compartments [14]. They are associated with secreted viral proteins such as HIV Nef protein, which differentially regulates the expression of Rab proteins and thus usurps vesicular trafficking to increase viral progeny [15]. Another component of vesicular transport that can be usurped is proteins of the caveolin family. Caveolin-1 (CAV-1) is an integral membrane protein that plays a key role in the formation of caveolae, flask-shaped invaginations in the plasma membrane involved in multiple cellular processes, such as endocytosis, signal transduction, and cholesterol and protein homeostasis. The dengue NS1 protein interacts with a complex formed by CAV-1 and the chaperones FKBP52, Cy40, and CyA to be secreted [16]. Interestingly, it has been suggested that the microtubules and actin filaments of rotavirus-infected cells participate in the secretion pathway of the NSP4 protein of rotavirus, which usurps these filaments to be secreted. This was demonstrated when cells were incubated with pharmacologic agents, such as Brefeldin (BFA), Nocodazole (NOC), and cytochalasin D (Cyt.D), and it was observed that inhibiting vesicular traffic through the network of microtubules and actin filaments blocked the secretion of NSP4 [17]. Phospholipids also play an important role in the secretion of viral proteins, such as phosphatidylinositol-4,5-bisphosphate (PI(4,5) P_2_), a phospholipid mainly located on the inner surface of the plasma membrane, where it acts as an anchor point for proteins involved in key cellular processes, including endocytosis, phagocytosis, exocytosis, and cell adhesion. PI(4,5) P_2_ recruits and interacts with the HIV Tat protein for secretion [18]. It is essential to mention that some viral proteins contain a signal peptide that enables them to use the classical protein secretion pathway that directs them through the ER and the Golgi apparatus to be subsequently secreted by exocytosis pathways, where multiprotein complexes such as COPI and COPII are involved. The viral protein ORF8 of SARS-CoV-2 uses this pathway to be secreted [19]. Secreted viral proteins usurp cellular pathways, such as extracellular vesicles, Rab proteins, caveolins, the cytoskeleton, and phospholipids, to be transported and secreted, in addition to using the classical secretion pathway, highlighting their ability to manipulate essential cellular processes to further propagate viral infection [11] (Figure 1).

## 3. Viral Secreted Proteins: Mechanisms and Relevance to Pathogenesis

### 3.1. Epstein–Barr Virus (EBV)

BamHI-A Rightward Frame 1 (BARF1)

The BARF1 protein has a molecular weight of approximately 33 kDa and can be found in both the cytoplasm and membrane of infected cells, from where it is secreted [20]. Structurally, the protein contains a combination of alpha helices and beta sheets, and its main domain is in the form of a beta-barrel. The beta-barrel is composed of multiple antiparallel beta strands that form a hydrophobic central core [21]. This protein contains a signal sequence in its N-terminal region, which directs its secretion. It has been found in the extracellular region in the form of a hexameric molecule stabilized by disulfide bridges [22]. As a key immunomodulator, it has been described as having important roles in the infection cycle. For example, it binds to macrophage colony-stimulating factor (M-CSF), inhibiting macrophage proliferation and activation, reducing inflammation, and limiting antigen presentation [23]. By activating pathways such as PI3K/AKT and MAPK/ERK, it induces changes that lead to the growth and survival of infected cells and the formation of carcinomas [24]. During type II latency, BARF1 suppresses immune responses, facilitates viral persistence, and helps maintain an immunosuppressive environment during reactivation. It favors angiogenesis and immune evasion in associated tumors, which are crucial in the pathogenesis and persistence of EBV [24]. Some studies have shown that this protein can transform cell lines, such as green monkey kidney epithelium and mouse fibroblasts [25]. Another relevant feature is that after being secreted, it is considered an oncoprotein as infected cells treated by chemotherapy and associated with gastric cancer can evade apoptosis signaling by increasing the B cell leukemia/lymphoma 2 (Bcl-2) pathway [26,27]. In gastric cancer, it has been shown that BARF1 can positively regulate the NF-κB/cyclin D1 signaling pathway, resulting in uncontrolled exacerbation of cell division, which, in turn, decreases the cell cycle inhibitor p21 [28]. Overall, BARF1 activates oncogenic pathways indirectly by altering extracellular signals such as M-CSF sequestration and inducing the activation of intracellular cascades (PI3K/AKT and MAPK/ERK). These pathways result in cell proliferation, survival, and transformation, contributing to the development of EBV-associated tumors such as nasopharyngeal and gastric carcinomas. BARF1 can turn on oncogenic pathways and turn off cell cycle inhibitors, thereby positioning this protein as a potential therapeutic target [29].

Viral Interleukin 10 (vIL-10 Protein)

EBV produces cellular cytokine homologs that facilitate the evasion or suppression of host antiviral responses, allowing EBV to establish a state of latency. Among these proteins, the viral interleukin 10 (vIL-10), the functional equivalent of human interleukin 10 (hIL-10), is notable. This protein has a molecular weight of approximately 17–21 kDa, depending on the post-translational modifications and experimental conditions used for its analysis [30]. Structurally, it shares similarities with hIL-10; it is a homodimeric protein, with each monomer composed of six alpha helices [31]. vIL-10 shares many key functions with its human counterpart, hIL-10, including inhibiting the production of inflammatory cytokines, such as IFNγ, promoting B cell proliferation and differentiation, and stimulating immunoglobulin production. However, there are also significant differences between hIL-10 and vIL-10. vIL-10v does not promote mouse thymocyte proliferation or mast cell proliferation, and it does not increase major histocompatibility complex (MHC) class II expression in B cells; these functions are unique to hIL-10. Furthermore, hIL-10 is essential for innate immunity, as the first line of defense against infections. In contrast, the role of vIL-10 in monocyte function is yet to be elucidated [32]. Some essential functions of vIL-10 in the infection cycle have been described. First, the protein is expressed in the early stages of infection to suppress the activation of innate immune cells, such as macrophages and dendritic cells, thereby inhibiting the presentation of antigens to the adaptive immune system. This allows the virus to avoid detection by cytotoxic T cells in the early stages of infection. It also plays a vital role in suppressing inflammation by inhibiting the production of proinflammatory cytokines, such as IL-1, IL-6, IL-12, IFN-γ, and TNF-α, which are crucial for coordinating an adequate inflammatory response against the virus. As the virus replicates, vIL-10 protects it from potential harm by the immune system. vIL-10 decreases the expression of major histocompatibility complex (MHC) class I molecules in infected cells, thereby preventing T cells and NK cells from detecting the presence of the virus [33]. Once secreted, vIL-10 binds to IL-10R1 and IL-10R2 receptors on immune cells, such as macrophages and dendritic cells, blocking the maturation of the latter and reducing their ability to activate T cells [33].

On the other hand, in systemic lupus erythematosus (SLE), vIL-10 can induce an imbalance in immune responses by the uncontrolled activation of immune cells. This could contribute to autoimmunity, as vIL-10 interferes with hIL-10 signaling, which usually has an inhibitory effect on inflammation and autoimmunity. This effect of vIL-10 on immune cells could be a key factor in the exacerbation of autoimmune responses in patients with SLE. Therefore, the relationship between vIL-10 and SLE could be mediated by altered cell signaling, leading to increased proliferation and activation of immune system cells, which may promote the tissue damage characteristic of this disease [34].

### 3.2. Dengue Virus

Nonstructural 1 Protein (NS1)

The NS1 protein of dengue virus has a molecular weight of approximately 40–50 kDa [35] and is structurally organized into three principal domains: the β-roll domain (N-terminal) that promotes dimerization and membrane interaction, the Wing domain (central), characterized by hydrophobic properties enabling anchorage to intracellular membranes, and the β-ladder domain (C-terminal) that confers structural stability and engages with the immune system. NS1 forms intracellular dimers and extracellular hexamers and is essential for viral replication and immune evasion [36]. The principal role of dengue NS1 in the infection cycle is its association with the replication complex, where it acts as a scaffolding protein that interacts with other viral proteins involved in this process, facilitating the spatial and functional organization of the essential components for viral RNA replication. NS1 localizes to membranes associated with the endoplasmic reticulum, where it interacts with viral proteins, such as NS4A and NS4B, and cellular factors to induce membrane remodeling and form characteristic vesicles that house the replication machinery [37,38]. In addition, this protein contributes to the stability of the replicative complex by coordinating the synthesis and processing of viral RNA, which ensures efficient and sustained replication of the viral genome [39]. However, NS1 is secreted via the classical pathway. In this pathway, NS1 is first translocated from the ER to the Golgi apparatus, where it is highly glycosylated to subsequently interact with the protein CAV-1, which is the main component of caveolae, and also participates in the transport of cholesterol, and with the chaperones FKBP52 and Cy40, ultimately enabling its secretion [3,5,39]. As mentioned previously, when secreted into the extracellular space, it can be found as hexamers (dimers, trimers). In this form, it has a disk-shaped structure [40]. One of the main functions of the protein, when secreted, is binding to spherical high-density lipoprotein (HDL) and low-density lipoprotein (LDL) particles, specifically in macrophages. This association can trigger proinflammatory signals favoring vascular permeability and viral dissemination, affecting endothelial permeability by binding to the glycocalyx, a protective layer of glycosaminoglycans and proteoglycans on the surface of endothelial cells. This interaction is focused on specific components such as heparan sulfate, which allows NS1 to anchor and trigger a series of detrimental effects [41]. Secreted NS1 (sNS1) activates enzymes, such as matrix metalloproteinases (MMPs) and heparanases, that degrade the structural components of the glycocalyx, weakening its function as a physical barrier and regulator of vascular flow [42].

Another important function of sNS1 is that it interacts with the complement system by activating the alternative pathway by binding to C3 and C5, which generates anaphylatoxins (C3a and C5a) that promote inflammation, cell recruitment, and vascular damage. At the same time, sNS1 inhibits the classical and lectin pathways by blocking key components, such as C1s, C1r, and MASPs, helping the virus to evade immune responses [43]. sNS1 is deposited on the endothelium, promoting local complement activation and membrane attack complex (MAC) formation, which amplifies vascular damage and permeability [44,45]. These interactions contribute to systemic inflammation, capillary leak syndrome, and immune evasion, exacerbating the pathogenesis of severe dengue [45]. On the other hand, when in its soluble form, sNS1 also promotes glycocalyx degradation, facilitating the leakage of plasma and serum proteins into the extravascular space, contributing to the edema and capillary leak syndrome characteristic of severe dengue [46]. In addition, NS1 induces inflammatory responses by activating receptors such as toll-like receptor 4 (TLR4) on endothelial cells, which stimulates the production of proinflammatory cytokines (IL-6, IL-8, TNF-α) and reactive oxygen species. This mechanism, which includes structural damage, oxidative stress, and inflammation, is key in the pathogenesis of severe dengue, especially in the development of capillary leak syndrome [47]. Clinically, sNS1 can serve as a good marker for early diagnosis as it has been reported to be present in levels exceeding 10 μg/mL in the blood [48,49,50].

### 3.3. Rotavirus

Nonstructural Protein 4 (NSP4)

Rotavirus NSP4 is a transmembrane glycoprotein with a molecular weight between 20 and 28 kDa [51]. It has several functional domains that allow it to play key roles in the viral replication cycle and pathogenesis. Structurally, NSP4 contains α-helices in its transmembrane domain, facilitating its ability to oligomerize in tetramers. In addition, it is composed of three hydrophobic domains, a coiled-coil domain, and a specific domain that allows its assembly into virus double-layer particles (DLPs), which constitute an intermediate stage in viral morphogenesis [52]. One of the most important functions of NSP4 is that it acts as a structural scaffold that facilitates the interaction between DLPs and the outer capsid proteins VP4 and VP7 [53]. This process is crucial for virus maturation, as it allows the incorporation of the outer coat, forming triple-layer particles (TLPs), which are the infectious forms of the virus. If this assembly process is not properly executed, virions cannot complete their replicative cycle or spread efficiently in the host [54]. In addition to its role in viral assembly, NSP4, the first viral enterotoxin to be characterized, plays a key role in the pathogenesis of rotavirus-induced diarrhea [55]. Its enterotoxin function is related to its ability to alter intracellular calcium levels in infected cells [41]. To exert this function, NSP4 must be released in its soluble form (sNSP4), which occurs when it is secreted by infected enterocytes. In its soluble form, sNSP4 can act on neighboring cells and modify calcium levels, triggering cytopathic effects [56].

Clinically, the enterotoxic action of NSP4 has been associated not only with severe diarrhea and dehydration in patients infected with rotavirus but also with the appearance of febrile convulsions in some cases. It has been proposed that this neurological manifestation could be related to the modulation of calcium levels in the central nervous system, which would affect neuronal activity and contribute to seizure episodes in children infected with rotavirus [57]. The mechanism by which NSP4 causes diarrhea is related to its ability to alter the ionic balance in intestinal epithelial cells. Its interaction with ion channels leads to increased calcium levels in the cytoplasm, which interferes with the function of ion transporters and causes uncontrolled water flow into the intestinal lumen, generating severe watery diarrhea, characteristic of rotavirus infection, which can lead to severe dehydration if not adequately treated [58,59]. Additionally, NSP4 acts as a viroporin, a viral protein class that alters cell membrane permeability. Here, NSP4 affects ion channels by modulating calcium and sodium levels, which exacerbates electrolyte and water loss in the intestine [60,61]. This viroporin activity reinforces the enterotoxic effect of NSP4, aggravating the severity of diarrhea in patients infected with rotavirus [62]. NSP4 plays a multifunctional role in rotavirus infection in viral replication and disease pathogenesis. Its ability to assemble infectious virions and alter ionic homeostasis in infected cells is key to viral dissemination and disease severity. Given its relevance in the pathophysiology of rotavirus, NSP4 remains a target of research interest for developing therapeutic strategies and vaccines that can mitigate the impact of this viral infection [63,64].

### 3.4. Human Immunodeficiency Virus (HIV)

Tat Protein (Tat)

Tat from HIV weighs approximately 14–16 kDa, with the size depending on the viral variant as there are shorter forms of Tat that have approximately 86 amino acids and some longer forms that have between 101 and 104 amino acids [65]. Structurally, Tat is considered highly disordered, and consists of both α-helices and β-sheets, which can change when the protein interacts with other molecules; therefore, it is also highly flexible [66]. One of the main functions of this protein is to optimize the efficiency of RNA polymerase II, allowing the generation of complete viral mRNAs [67]. Tat interacts with a looped RNA sequence located at the 5’ end of RNA; this sequence is named the transactivation response element (TAR) [68]. Its activity mainly depends on association with the cellular cofactor cyclin T1 (CCNT1), which, together with cyclin-dependent kinase 9 (CDK9), forms positive transcriptional elongation factor b (P-TEFb). This host complex phosphorylates essential residues of paused RNA polymerase II, facilitating its activation and ensuring the efficient progression of transcription. Initially, it was proposed that Tat recruits the P-TEFb complex to the nascent RNA TAR, positioning the kinase close to the paused polymerase for activation via phosphorylation. Moreover, CCNT1 is a linker between Tat and CDK9 and interacts directly with TAR RNA through base contacts in its loop, ensuring high-affinity binding [69]. The formation of the Tat–P-TEFb complex significantly potentiates the transcription of the viral genome, driving post-integrative stages in the HIV-1 life cycle and promoting the production of new viral particles, thus enabling the spread of infection to uninfected cells [70]. A significant amount of Tat produced by the infected cell is released into the extracellular milieu without the occurrence of cell death or alterations to membrane permeability. This release generates a local reservoir of extracellular Tat, which plays a critical role in the establishment and spread of HIV infection [71]. Once in the extracellular environment, Tat binds across the basic region to heparan sulfate proteoglycans (HSPGs) present in the extracellular matrix, forming persistent chemotactic gradients that attract HIV-1 target cells, such as monocytes, macrophages, and activated endothelial cells, to the site of infection [72]. In addition, extracellular Tat is highly efficiently taken up by activated dendritic cells and endothelial cells and, to a lesser extent, by CD4^+^ T lymphocytes, where it activates the expression of genes that promote viral shedding. In naïve CD4^+^ T cells, Tat increases the expression of the HIV-1 co-receptors CXCR4 and CCR5 while blocking the function of CXCR4, which increases the susceptibility of these cells to infection by HIV-1 variants with R5 tropism [73,74]. It also induces an alternative activation pathway in these cells, making them more prone to productive infection [51]. On the other hand, extracellular Tat associates with the HIV-1 trimeric envelope glycoprotein (Env), forming a complex that facilitates viral entry through RGD-binding integrins, which are highly expressed in dendritic cells. This mechanism enhances the infection of these cells, promoting their maturation toward a phenotype that favors the T helper 1 (Th1) immune response. Likewise, Tat stimulates the production and release of inflammatory cytokines in different cell types, which activate endothelial cells and make them more susceptible to virus entry and productive infection. Furthermore, the binding of extracellular Tat to the membrane HSPG of infected cells can induce apoptosis of effector CD8^+^ T lymphocytes, contributing to the immune evasion of HIV [75].

### 3.5. Respiratory Syncytial Virus (RSV)

Glycoprotein (G)

The RSV glycoprotein has an approximate weight of 90 kDa, which can vary depending on the degree of glycosylation [76]. Structurally, it is a highly glycosylated membrane protein that plays a key role in the adhesion of the virus to host cells. It has three main domains: an extracellular domain responsible for interacting with cellular receptors, a hydrophobic transmembrane domain anchoring the protein to the viral membrane, and a short cytoplasmic domain [77]. The extracellular domain is rich in serine and threonine residues, which are O-glycosylation sites and contain N-glycosylation sites. This domain is characterized by a conserved central region with a heparan sulfate motif that facilitates binding to host cells. Glycoprotein G is found both as a membrane protein and in a secreted (soluble) form, which acts as a decoy to divert the host’s immune response [77]. The soluble form of RSV glycoprotein G (sG) is generated mainly by proteolytic cleavage of its membrane form. During infection, cellular proteases, such as MMPs, cleave the extracellular region of glycoprotein G, releasing it into the extracellular environment. This process, known as shedding, allows sG to interfere with the immune response by acting as a decoy for antibodies and altering chemokine signaling, thereby contributing to RSV pathogenesis [78]. sG plays multiple roles that promote viral infection and contribute to pathogenesis. It modulates the innate immune response by inhibiting pattern recognition receptors, such as toll-like receptors (TLR4), thereby reducing the production of proinflammatory cytokines and allowing the virus to evade early detection by the immune system. Furthermore, it inhibits the chemotaxis of immune cells, such as monocytes, macrophages, and neutrophils, blocking their migration to infection sites and decreasing the host’s ability to control viral replication. sG acts as an immunological decoy by binding to specific antibodies, reducing their neutralizing efficacy, and thereby creating a favorable environment for viral persistence [79]. It also retains the ability to interact with heparan sulfate receptors on host cells, altering cellular functions and local regulations of the immune response [80]. On the other hand, although sG can suppress inflammation in specific contexts, it can also exacerbate inflammation by inducing the release of proinflammatory cytokines, which aggravate respiratory epithelial damage and contribute to the clinical symptoms characteristic of RSV infection. These functions make sG a key factor in immune evasion, dysregulated inflammation, and promoting disease-associated tissue damage [78,79,80,81,82,83]. Interestingly, it has been shown in murine models that the presence of sG can enhance the cytotoxic response of CD8^+^ T cells. This suggests a possible advantage in terms of a protective response against RSV [84]. However, this response is not without risks, as, in conditions of an exacerbated immune response, increased cytotoxicity can contribute to worsening symptoms, leading to deterioration of the host’s condition [84]. Additional evidence of this phenomenon comes from trials performed in murine models, in which mice were immunized with glycoprotein G. The analysis of these animals showed exacerbated inflammation in the lungs, associated with a high production of proinflammatory cytokines, such as TNF-α, IL-6, and IL-1β [84]. This inflammatory environment can worsen tissue damage and contribute to severe respiratory dysfunction [85].

### 3.6. SARS-CoV-2

Open Reading Frame 8 (ORF8)

The SARS-CoV-2 accessory protein ORF8 has a molecular weight of approximately 17 kDa. Structurally, it has an immunoglobulin-like folded central domain (Ig-like fold), formed mainly by antiparallel beta sheets and stabilized by a disulfide bridge between two highly conserved cysteines (Cys20 and Cys37) [86]. In addition, it contains disordered regions at the N- and C-terminals, and its flexible and dynamic structure favors interactions with various host molecules, contributing to immune evasion and modulation of the inflammatory response [87]. ORF8 interacts with the protein Calnexin, an endoplasmic reticulum (ER) resident chaperone involved in proper glycoprotein folding and ER quality control. ORF8 can bind Calnexin through its ability to modify glycosylation and protein folding pathways, which could affect ER homeostasis and contribute to ER stress in infected cells. This interaction has also been linked to the ability of ORF8 to interfere with MHC class I (MHC-I), thereby modifying the efficiency of antigen presentation, facilitating evasion of the immune system by the virus [88]. On the other hand, it has been described that ORF8 can be secreted simultaneously by both conventional and unconventional secretion pathways. However, when secreted via the unconventional pathway, it may have an association with the interleukin 17 receptor type A (IL17RA), a key component in the cytokine-mediated inflammatory response, specifically in the activation of the IL-17 signaling pathway [88]. The interaction between ORF8 and IL17RA suggests that ORF8 could modulate the host immune response, particularly inflammation. It has been observed that ORF8 can induce an exacerbated and dysregulated inflammatory response by interacting with IL17RA, which may contribute to the immune dysfunction and tissue damage observed in severe COVID-19 infections [88]. This interaction could also be related to the increased production of proinflammatory cytokines, such as IL-6, TNF-α, and IL-1β, associated with severe forms of the disease [89]. This uncontrolled cytokine production generates a vicious cycle of inflammation known as a “cytokine storm”, which can cause severe damage to tissues such as the lungs, resulting in acute respiratory distress syndrome (ARDS) and other serious complications in COVID-19 [90].

In addition, ORF8 has been shown to interact with C3b, one of the key proteins of the complement system, which is responsible for immune response against pathogens. C3b plays an important role in the opsonization of pathogens and activation of the classical and alternative complement pathways. The interaction of ORF8 with C3b could interfere with the complement system, deregulating the normal activation of this immune pathway [91]. It has been proposed that ORF8 may inhibit the formation of membrane attack complexes or reduce the effectiveness of the opsonization of infected cells, facilitating immune evasion of the virus. This modulation of complement activity could contribute to the persistence of the virus in the body and exacerbation of inflammation in severe cases of COVID-19, as complement dysfunction has been linked to an increase in uncontrolled immune response and systemic inflammation [92]. In animal models, ORF8 attenuation reduces viral progeny and lung damage. Although ORF8 has a low level of homology with ORF7, both share a signal peptide [93]. Recently, it was determined that ORF8 crystallizes as a covalent dimer with multiple disulfide bonds and a structure formed by β-sheets [94]. In addition, studies have demonstrated its extracellular secretion and correlation with the severity of infection in patients, highlighting its importance for the development of new therapies, such as specific monoclonal antibodies [94,95].

Nonstructural Protein 2 (NSP2)

NSP2, also from SARS-CoV-2, has a molecular weight of approximately 70–80 kDa. It is structurally complex with multiple domains—a Zinc-binding site in the N-terminal domain and beta sheets in the C-terminal domain. It has a four-leaf clover-like conformation [96]. The functions of this protein have not yet been elucidated. Mass spectrometry studies and affinity assays suggest that it can interact with Grb10 Interacting GYF Protein 2 (GIGYF2), a cellular protein involved in mRNA regulation. NSP2 interacts with the GIGYF2/4EHP complex to inhibit the translation of *IFNB1* mRNA, thus blocking the production of IFNB, one of the most important molecules involved in the antiviral innate immune response. This mechanism is shared with SARS-CoV-1 and other RNA viruses such as VSV. Unlike NSP1 and NSP14, which globally inhibit host mRNA translation, NSP2 acts specifically through GIGYF2/4EHP, possibly affecting other antiviral cytokines. The LHR region of GIGYF2 is key in this interaction. Understanding this could guide the development of inhibitors aimed at disrupting NSP2 action and strengthening the antiviral immune response [97]. Moreover, NSP2 has also been reported to interact with prohibitin-1 (PHB1) and prohibitin-2 (PHB2), key proteins in mitochondrial organization, cell signaling, and translation regulation. This interaction occurs in the mitochondria and cytoplasm, altering essential functions. NSP2 could affect the stability of the mitochondrial membrane, reduce ATP production, and promote mitochondrial stress. It may also modify autophagy and vesicular trafficking, facilitating the formation of compartments favorable for viral replication. Furthermore, by sequestering PHB1/PHB2, NSP2 deregulates signaling pathways such as mTOR and Ras/MAPK, suppressing antiviral responses. These alterations suggest that the virus reconfigures the host cell to optimize its replication, making PHB1 and PHB2 potential therapeutic targets to limit infection [96,97]. NSP2 appears to be secreted through an unconventional pathway and can cross the plasma membrane of endothelial and tumor cells, localizing in the cytoplasm upon entry. Its internalization depends on actin polymerization as cytochalasin D inhibits this process. In addition, NSP2 can transport other molecules into the cell, functioning as a protein delivery vehicle. An experiment with the NSP2–BLF1 fusion protein showed that it translocates in Huh7 cells and causes cell death, like the action of *Burkholderia pseudomallei* lethal factor 1 (BLF1), which inhibits the translation initiation factor eIF4A and possesses antitumor activity. Different studies, such as protein purification, chemical inhibition, and cellular imaging, have confirmed that NSP2 facilitates the delivery of heterologous proteins in human cells [97]. These properties suggest a role for NSP2 in modulating the cellular environment and open the possibility of using NSP2 as a therapeutic delivery system for proteins with specific functions, such as toxins or antiviral agents. In addition, the ability of NSP2 to enter tumor cells could indicate its potential in targeted therapies, similar to other viral proteins that alter cell biology [97].

### 3.7. Ebola Virus

#### Delta Peptide (Δ-Peptide)

The delta peptide is a nonstructural peptide of approximately 40 amino acids with a molecular weight between 10 and 14 kDa [98]. It is formed as a result of the post-translational events of the soluble glycoprotein (sGP) and, like the sGP, is secreted by cells infected by this virus [98,99]. Its structure has not been established; however, studies have shown that it contains alpha helices and behaves as an amphipathic peptide as it has hydrophobic regions and polar charges. This characteristic allows it to insert itself into cell membranes and alter their permeability, which makes it an important factor in viral pathogenesis [99].

One of the most prominent functions of the delta peptide is the alteration of the ionic permeability of cell membranes by acting as a viroporin. This is achieved by forming pores in membranes through the interaction of highly conserved cysteine disulfide bonds. These pores induce osmotic imbalance and may contribute to the disruption of essential cellular processes, favoring viral replication and evasion of the host immune response [100].

In addition, the delta peptide has been found to share functional similarities with the viral enterotoxin NSP4 of rotavirus, which is particularly interesting from a comparative perspective. Both molecules are rich in cationic and aromatic residues [100]. This protein could induce membrane permeabilization, suggesting a possible convergent evolutionary mechanism, in which different viruses have evolved similar strategies to induce diarrhea and facilitate their propagation in the host [101]. Clinical studies showed that in patients infected with Ebola, significantly lower levels of delta peptide than glycoprotein G of the virus have been detected; however, experimental studies have shown that delta peptide possesses significant enterotoxigenic activity. For example, in a murine ileal ligated loop model previously used to study the effect of *Vibrio cholerae*, different concentrations of the peptide in its oxidized form (E23 ox) were injected, and an analysis of the diarrheal response over time showed a moderate fluid secretion at 6 h, and peaking between 9 and 12 h. This phenomenon was accompanied by intestinal distension, vascularization, and the accumulation of cellular debris, suggesting a direct cytotoxic effect on the intestinal epithelium [102].

**Figure 2:**
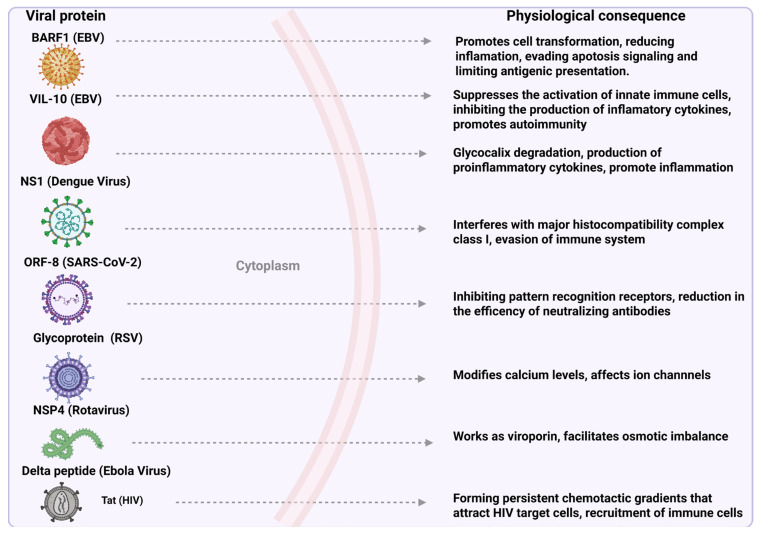
Basic functions of viral proteins once secreted into the extracellular medium. It has been reported that during infection by viruses such as EBV, dengue virus, SARS-CoV-2, RSV, rotavirus, Ebola virus, and HIV, some proteins are secreted into the extracellular medium, where they play an important role in concluding the cycle of viral pathogenesis. Some of these proteins are BARF1 and VIL-10 (EBV), NS1 (dengue virus), ORF-8 (SARS-CoV-2), glycoprotein (RSV), NSP4 (rotavirus), delta peptide (Ebola virus), and Tat (HIV). These proteins can act as immunomodulators, inhibiting the activity of immune cells or inducing the production of immunosuppressive cytokines. Others can interfere with cell signaling pathways, leading to dysregulation of cellular processes and increased viral replication.

The activity of the delta peptide in the gastrointestinal system could be mediated by the alteration of ion channels and increased paracellular permeability, which is a mechanism similar to that observed in other viral and bacterial toxins. These findings raise questions about its possible involvement in the systemic dissemination of the virus, as well as in the development of gastrointestinal complications in patients infected with Ebola. Future research could focus on characterizing the three-dimensional structure of the delta peptide using advanced techniques, such as nuclear magnetic resonance (NMR) or X-ray crystallography, to better understand its mechanism of action and its potential as a therapeutic target in the treatment of viral infections [103]. Finally, it is relevant to highlight that the secretion of peptides such as delta peptides not only contributes to the pathogenesis of the Ebola virus but could also have immunomodulatory implications. Recent studies have suggested that these peptides could interfere with local immune signaling, facilitating immune evasion and promoting viral persistence [104]. Therefore, a detailed study of the delta peptide and its interaction with cell membranes could open new avenues for the development of therapeutic interventions aimed at blocking its activity and reducing the severity of clinical manifestations associated with Ebola virus infection [105] (Figure 2).

## 4. Emerging Technologies for Viral Diagnostics Based on Secreted Proteins

The diagnosis of viral infections remains a challenge today as there are multiple factors that can affect the accuracy of previously established algorithms for pathogen detection. One of the main challenges lies in the ability of viruses to constantly evolve through genetic mutations [106,107]. These modifications can affect key regions of the viral genome, which in turn compromises the sensitivity and specificity of molecular tests that are based on nucleic acid detection. A clear example of this phenomenon is SARS-CoV-2, the virus responsible for the COVID-19 pandemic. Throughout its evolution, this virus has accumulated several mutations that have impacted its biology and, in some cases, the effectiveness of diagnostic tests. For example, the Alpha variant (B.1.1.7) had a deletion in the S gene region (Δ69–70), which affected the detection ability of certain RT-PCR tests targeting that segment. Similarly, the Omicron variant (B.1.1.529) incorporated numerous mutations in the Spike protein, some of which also caused failures in assays designed for specific sequences [108]. These alterations highlight the need to continuously update and adapt diagnostic methods, ensuring that they can accurately detect emerging variants and minimize the risk of false negatives. Furthermore, they underline the importance of genomic surveillance to monitor the evolution of viruses and its impact on diagnostic tools [108].

The differential diagnosis of viral infections represents a significant challenge owing to the similarity in signs and symptoms presented by patients with different infections. A clear example of such a situation is infections caused by arboviruses, such as dengue, Zika, and Chikungunya, which present with similar clinical manifestations, including fever, rash, arthralgias, and myalgias [109]. This overlap of symptoms can make accurate identification of the causative agent difficult, which in turn can delay appropriate treatment and epidemiological control measures [110]. Therefore, the need to develop and apply highly specific diagnostic methods that can accurately differentiate between these viruses becomes evident. Although molecular and serological tests have advanced significantly, there are still limitations in terms of specificity and sensitivity, especially in regions where co-circulation of multiple arboviruses is frequent [111]. A promising alternative diagnostic method is the identification of secreted viral proteins as potential biomarkers for both diagnosis and prognosis of the disease. Some viral proteins may play a key role in modulating the host immune response and pathogenesis, making them viable candidates for improving the early detection and stratification of patients according to the severity of infection [111].

For example, in the case of dengue, NS1 has proven to be a useful marker not only for early diagnosis, but also as a possible prognostic indicator of disease severity. Similarly, identifying proteins secreted in other viral infections could contribute to optimizing diagnostic accuracy and allow a better differentiation between pathogens with similar clinical presentations [112]. In a clinical study, the amount of detectable ORF8 protein in the serum of patients with SARS-CoV-2 infection was correlated with the severity, where a higher level of ORF8 was associated with greater symptom severity and vice versa [113]. The development of tools based on these viral proteins could improve clinical decision making, enabling more timely and effective treatments. In addition, these biomarkers could complement traditional tests, providing additional information on disease progression and facilitating a more personalized medical response [113].

The conventional diagnosis of viral diseases is mainly based on the detection of antibodies specific to proteins of the pathogen. However, secreted viral proteins, which are actively released by infected cells and found in the systemic circulation, represent a potentially useful target for developing new diagnostic tools. The identification and quantification of these proteins could facilitate the early detection of infection, improve diagnostic accuracy, and potentially provide prognostic information [114]. Some assays, such as surface plasmon resonance (SPR), could be used for the diagnosis of viral diseases. This technique is based on the measurement of changes in the refractive index on the surface of a sensor coated with a specific ligand (such as antibodies directed against viral proteins). For example, the antigen NS1 was employed as a detection target through its interaction with an anti-NS1 antibody, resulting in a variation in the refractive index of the sensor. The SPR technique showed a sensitivity comparable to that of conventional diagnostic methods; however, the SPR technique offered advantages in terms of efficiency and simplicity in diagnosing infection. These findings highlight SPR as a promising tool for the detection of dengue virus (DENV) and, potentially, other viral diseases [114,115]. The SPR system has several advantages compared to traditional methods such as ELISA and RT-PCR. First, SPR offers superior sensitivity, allowing the detection of minute amounts of NS1 antigen (up to 0.300 nM), which is difficult with other methods. In addition, its accuracy is higher owing to the higher refractive index than the optical density of ELISA. Unlike RT-PCR and ELISA, SPR is more time efficient, as it does not require long amplification or incubation steps. It is also less labor-intensive and does not require highly specialized personnel, simplifying its application in clinical settings [115]. Another key advantage is that anti-NS1 antibodies immobilized on the sensor surface can be reused multiple times, which reduces costs and optimizes resources. Although lateral flow assays are fast, their reliability in detecting NS1 is lower than that of SPR. Although further studies are still required to evaluate its performance in different regions and with other flaviviruses, SPR is emerging as a viable commercial alternative with potential applications in the diagnosis of various diseases [115].

**Figure 3:**
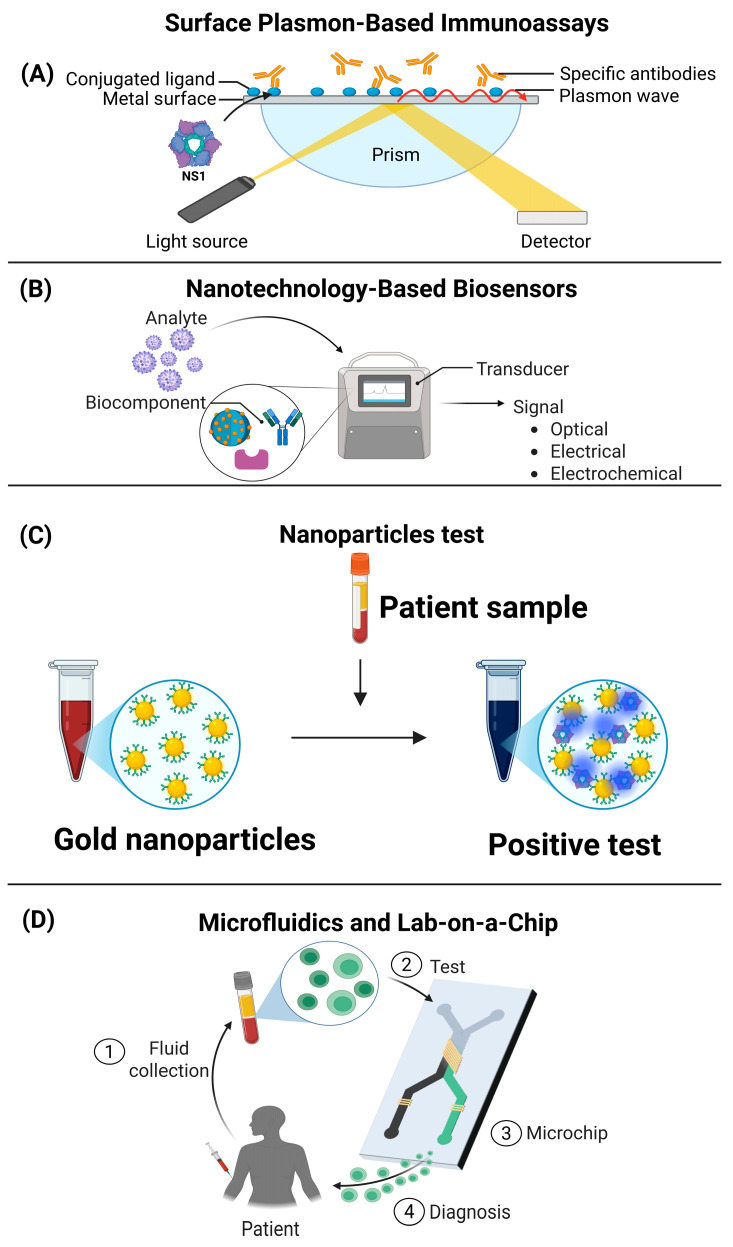
Viral diagnostic techniques are based on soluble viral proteins. (**A**) Surface plasmon resonance immunoassay-based: This technique is used for the specific detection of antibodies against soluble proteins in patient samples such as serum or saliva. Where proteins such as NS1 are fixed on the metal surface, and subsequently the sample is passed where specific antibodies bind to the antigen, and a light beam will be incident where the plasmons emitted by the prism will be detected in an electronic detector. (**B**) Biosensors: This technique is based on the analysis of biological samples through electronic equipment that detects soluble proteins, which are translated into optical, electrical, and electrochemical signals, and these signals can be quantified. (**C**) Gold nanoparticle colorimetric test: This technique is based on the coating of gold particles with antibodies specific to viral proteins, whose sensitivity can be used to evaluate patient samples, where the antigen-antibody interaction translates into a color change in the solution to the problem. The colorimetric change represented by the arrow (**D**) Microchips: This technique, where protein detection processes are designed in very small structures and where small quantities of biological fluids can be evaluated, is highly effective, allowing its widespread use in clinical studies. 1. The sample can be taken from patient serum by taking blood, 2. The serum is recovered from the blood and this is added directly to the microchip, 3. The microchip recognizes the viral antigens that need to be detected, 4. Based on the quantity and presence of these antigens, a disease caused by these microorganisms can be diagnosed and predicted.

Electrochemical biosensors can also be used for the diagnosis of viral proteins. These biosensors are devices or tools that detect changes in electrical current upon binding of a specific viral protein, and these sensors could be integrated into point-of-care (PoC) testing devices similar to a glucometer. There are reports of biosensors programmed for the diagnosis of SARS-CoV-2 specifically fused to the ACE2 protein; however, being programmed biosensors, they can be fused to any viral protein, including secreted proteins. The main biofluids that can be analyzed in these systems include the urine, saliva, and serum of patients [116]. Biotechnological applications have significantly increased in recent years in medicine, including in the diagnosis of viral diseases. Coupled gold nanomolecules coated with antibodies or aptamers directed against secreted viral proteins could be integrated into portable devices for rapid detection in serum or plasma. Some tools have fused gold nanoparticles to monoclonal antibodies for the detection of the viral protein hemagglutinin of Influenza virus type A, developed by Li and collaborators [115,116]. Further, a colorimetric assay has also been developed based on the association of the main protease of SARS-CoV-2 (Mpro) with gold nanoparticles (AuNPs), where the intensity of color change is directly related to the electrostatic interaction [117]. Finally, some advanced optical lab-on-a-chip microfluidic biosensors allow the separation and detection of viral proteins from minimal sample volumes. These devices could combine immunocapture with mass spectrometry to identify multiple secreted viral proteins simultaneously. An optical biosensor essentially translates virion capture into a measurable alteration of a property of light, such as refractive index (IR), intensity, or resonance changes, through different methods such as resonators and interferometers. Therefore, optical biosensors can be coupled with different molecules, such as proteins, antibodies, RNA, peptides, and DNA, which makes them very useful in the diagnosis of viral diseases [118,119] (Figure 3).

## 5. Conclusions

Secreted viral proteins are tools that viruses use to strategically interact with host cells and evade the immune system. Secreted proteins, such as BARF1 (Epstein–Barr), NS1 (dengue), and ORF8 (SARS-CoV-2), play crucial roles in pathogenesis and inflammatory modulation, the control of apoptosis, and the alteration of cellular homeostasis. Studies have revealed the importance of secreted proteins in viral, autoimmune, and oncological diseases. Moreover, they are also promising targets for the development of vaccines, antiviral therapies, and diagnostics. Proteins such as NS1 from dengue, NSP4 from rotavirus, and Tat from HIV are key in diagnostics and immunotherapies, while others, such as ORF8, are associated with the severity of infections, opening possibilities for targeted therapies. Secreted viral proteins represent a promising alternative for the early and accurate diagnosis of viral diseases. Emerging technologies, such as surface plasmon resonance, electrochemical biosensors, and microfluidic devices, have demonstrated potential to detect these proteins in various biofluids. The integration of these tools into portable devices could facilitate rapid and accessible diagnostics. Thus, the development of these innovative techniques could significantly transform the detection and management of viral infections.

## Data Availability

No new data were created or analyzed in this study.
